# Global Health Education: a cross-sectional study among German medical students to identify needs, deficits and potential benefits (Part 2 of 2: Knowledge gaps and potential benefits)

**DOI:** 10.1186/1472-6920-10-67

**Published:** 2010-10-08

**Authors:** Kayvan Bozorgmehr, Johannes Menzel-Severing, Kirsten Schubert, Peter Tinnemann

**Affiliations:** 1 Institute for Social Medicine, Epidemiology and Health Economics, Charité - University Medical Center, Berlin, Germany; 2 Globalisation and Health Initiative (GandHI), German Medical Students' Association (bvmd), Bonn, Germany

## Abstract

**Background:**

In Germany, educational deficits or potential benefits involved in global health education have not been analysed till now.

**Objective:**

We assess the importance medical students place on learning about social determinants of health (SDH) and assess their knowledge of global health topics in relation to (i) mobility patterns, their education in (ii) tropical medicine or (iii) global health.

**Methods:**

Cross-sectional study among medical students from all 36 medical schools in Germany using a web-based, semi-structured questionnaire. Participants were recruited via mailing-lists of students' unions, all medical students registered in 2007 were eligible to participate in the study. We captured international mobility patterns, exposure to global health learning opportunities and attitudes to learning about SDH. Both an objective and subjective knowledge assessment were performed.

**Results:**

1126 online-replies were received and analysed. International health electives in developing countries correlated significantly with a higher importance placed on all provided SDH (p ≤ 0.006). Participation in tropical medicine (p < 0.03) and global health courses (p < 0.02) were significantly associated with a higher rating of 'culture, language and religion' and the 'economic system'. Global health trainings correlated with significantly higher ratings of the 'educational system' (p = 0.007) and the 'health system structure' (p = 0.007), while the item 'politics' was marginally significant (p = 0.053).

In the knowledge assessment students achieved an average score of 3.6 (SD 1.5; Mdn 4.0), 75% achieved a score of 4.0 or less (Q_25 _= 3.0; Q_75 _= 4.0) from a maximum achievable score of 8.0. A better performance was associated with international health electives (p = 0.032), participation in tropical medicine (p = 0.038) and global health (p = 0.258) courses.

**Conclusion:**

The importance medical students in our sample placed on learning about SDH strongly interacts with students' mobility, and participation in tropical medicine and global health courses. The knowledge assessment revealed deficits and outlined needs to further analyse education gaps in global health. Developing concerted educational interventions aimed at fostering students' engagement with SDH could make full use of synergy effects inherent in student mobility, tropical medicine and global health education.

## Background

Our increasingly globalised world adds to the complexity of health and its determinants. Debates on the health impacts of globalisation are controversial, but it is acknowledged that globalisation has enabled people to engage with each other in 'one world' by reducing barriers through 'supraterritorial processes', whose impacts however always 'touch down' in territorial localities [1]. A good example of these processes and the reduction of barriers is the increasing mobility of medical students during their studies, with an increasing proportion completing their electives abroad. This is the case for students in the United States of America [2] and the United Kingdom [3] and, as shown in part one of this series, has been the case for the majority of German graduating students among our sample [4].

It is noteworthy that the reduction of physical, legal, cultural and psychological barriers has not only influenced the spread of diseases and risk-factors within and between populations, but has also re-emphasised the role of the social determinants of health (SDH) in understanding and tackling root causes of ill health and health inequities within and across countries.

The term 'social determinants' is thus shorthand for the social, political, economic, environmental and cultural factors that greatly affect health status [5]. Strong linkages between globalisation and health have been demonstrated and evidence-informed policy recommendations for action on the SDH have been recently formulated by the Commission on the Social Determinants of Health (CSDH) for local and global purposes [6]; reinforcing the role of primary health care as the global strategy to universal coverage and health for all [7]. Acknowledging the role of health professionals in reducing health inequities on regional, national, international and global levels [6], the commission recommended in its final report to the World Health Organisation (WHO):

"Educational institutions and relevant ministries make the social determinants of health a **standard and compulsory **part of training of medical and health professionals." (*Recommendation 16.5)*

Strong evidence points to the influences of SDH on people's health, which legitimates the recommendation that medical and other health professionals should learn about them [6]. Although these influences mostly extend beyond the reach of "prescriptions" and "clinical interventions", knowing about them is a prerequisite for health professionals to address the root causes of ill health via societal action and public health or health promotion interventions.

In Germany, learning about the SDH has been mandatory in medical curricula since the early 1970s. The implementation of public health issues into medical curricula however has too often been criticised as insufficient [8-10]. With a recent reform of the Licensing Regulations [11] so-called cross-sectional subjects (*Querschnittsbereiche*) have been introduced into medical curricula [12,13] and have upgraded mandatory teaching in public health issues in quantitative and qualitative terms.

However, it has been shown that medical students lack an interest in learning about the SDH and that they regard the subjects which impart knowledge of the SDH as not relevant for their work [14,15].

Although the recommendation of the CSDH provides institutional backup of the importance of learning about the SDH for the health workforce, it remains yet unclear which factors (beyond adaptations in teaching methodologies [16,17]) could enforce an interest among medical students - and thus among future health professionals - to learn about the non-biomedical causes of ill health. Expert opinion states that the reduction of barriers in physical, legal, cultural and psychological aspects, which is for instance reflected in the high mobility of medical students during their studies, entails a potential to teach medical students about health and its (social) determinants in a global dimension *(Rowson M, Hughes R, Smith A, Maini A, Martin S, Miranda JJ, Pollit V, Wake R, Willott C, Yudkin JS: Global Health and medical education - definitions, rationale and practice, unpublished)*.

In this context it is important to note that 40 years after their introduction into medical education, socio-medical subjects in Germany deal with the SDH predominantly in a national context. A systematic review of subject catalogues for socio-medical teaching in Germany identified only 11 topics with explicitly international or global character among more than 300 core topics *(Bozorgmehr K, Tinnemann P: The State of Global Health in German Medical Education: a systematic review, unpublished)*. Evidence produced by this review further suggests that contemporary teaching, not only in socio-medical subjects, but also in tropical medicine/international health neglects important transborder and global determinants of health. International health courses exist in curricula as an optional offer, but mainly focus on the bio-medical aetiology, bio-medical diagnosis and bio-medical treatment of infectious diseases in the tropics, while the broader socio-political context of public health in developing countries is subordinated - 15 years ago *(Stich A, Köbler C, Strauß R, Hampel D, Fleischer K: Tropenmedizinische Ausbildung in Deutschland - Erfolge und Defizite: Teil 1-Lehrveranstaltungen zum Themenbereich "Tropenmedizin und Gesundheitsversorgung in Entwicklungsländern" an deutschen medizinischen Fakultäten, unpublished)*, but also presently *(Bozorgmehr K, Tinnemann P: The State of Global Health in German Medical Education: a systematic review, unpublished)*.

Findings presented in the first part of this article [4] suggest that some topics related to 'the influence on people's health of factors such as poverty, debt, globalisation, health systems and health financing, human rights, hunger, armed conflicts and migration' may be covered at various medical schools, e.g. integrated into compulsory courses or provided by students [4]. Self-contained and structured courses in global health, however, which explicitly and more comprehensively deal with the broader determinants of health on a global scale are rather offered as part of non-formal education, either organised by student organisations [18], other non-governmental organisations or as extra-curricular options in summer schools.

The gap in addressing international or global dimensions of SDH in formal medical education raises questions about students' knowledge of these. However, beyond expert opinions it remains unclear what medical students in Germany know about these or other global health issues. In an attempt to gather evidence on educational outcomes in this context within the published educational research literature in Germany, we could identify only one objective assessment of students' knowledge eligible to be considered as relevant to teaching in public, international or global health *(Bozorgmehr K, Tinnemann P: The State of Global Health in German Medical Education: a systematic review, unpublished)*. The identified work revealed knowledge gaps among medical students related to the epidemiology of tuberculosis and tetanus [19] and focused only on the aggregated prevalence in Germany rather than on the social epidemiology of those diseases; not to mention the importance for public health globally. No studies could be found which directly assessed students' knowledge of global health issues. Although the German Licensing Regulations for physicians [11] emphasise the importance of the population perspective as an integral part of medical studies, the evidence base on educational outcomes of German medical education related to socio-medical issues is poor; especially on those directly related to international or global health issues *(Bozorgmehr K, Tinnemann P: The State of Global Health in German Medical Education: a systematic review, unpublished)*.

### Purpose of this study

We aim to assess the importance medical students place on learning about selected social determinants of health (SDH), explore their knowledge of selected global health topics and analyse any associations with medical students' (i) mobility patterns and education in (ii) tropical medicine or (iii) global health.

## Methods

### Study Design

Nationwide, cross-sectional study conducted between May and December 2007 by the Globalisation and Health Initiative (GandHI) of the German Medical Students' Association (*Bundesvertretung der Medizinstudierenden e.V*.) (bvmd). We created a web-based, semi-structured questionnaire with 28 questions (25 structured, 3 open) in German language. The questions addressing the purposes of this paper captured the importance placed on learning about selected SDH, student demographics, experiences abroad, destination countries and exposure to relevant educational interventions (i.e. previous participation in courses of tropical medicine or global health). The Questionnaire Outline describes the questions related to the SDH and to the knowledge assessment in details.

The survey was anonymous and participants gave their informed consent for participation. The answers and the identity of the respondents could not be connected. Ethical approval for this study was exempt according to section (§) 15 of the professional code of conduct of the Medical Council of Berlin, Germany.

### Recruitment

Using electronic mailing-lists of German students' unions, medical students from all 36 German medical schools were invited by e-mail to complete the online-survey. In addition, internet links were established on the website of the German Medical Students' Association http://www.bvmd.de and on the website of a German medical students' journal http://www.aerzteblatt-studieren.de/. All registered medical students were eligible to participate in the study, but registration validity was not checked before filling in the online questionnaire. The call for participation simply contained a contextual reference to issues of medical education. To reduce selection-bias we avoided any words in the announcement which could be associated with global health, globalisation, development aid, development cooperation, international health or public health.

### Questionnaire Outline

The questionnaire consisted of five different blocks of questions. Three blocks have been described previously [4] and referred to students' mobility (i.e. experiences abroad; destination countries) and their previous exposure to relevant educational interventions (i.e. participation in courses of tropical medicine or global health). The questions referring to the SDH and to the knowledge assessment are presented in this paper, partially with the original German wording added in brackets.

#### The importance placed on learning about the social determinants of health

To capture the importance placed on learning about the social determinants of health (SDH), students were asked:

"Regardless of whether you have been abroad yet or not: How important is it for you to learn about the following factors prior to an international health elective?". (*Egal ob Du im Ausland warst oder nicht, wie wichtig findest Du es sich vor einer Famulatur oder PJ im Ausland über die folgenden Dinge zu informieren?*)

Subsequently, respondents were given five SDH and brief explanations on the respective determinants in brackets. These consisted of:

- the "educational system (accessibility, private/public funded, illiteracy rates,...)",

- the "economic system (gross national product, income distribution, trade agreements, debts,...)",

- "politics (good governance, stability,...)",

- "health system structure (private/public funded, infrastructure/facilities, health personal training,...)" and

- "culture, language and religion".

Students were asked to rate the importance of learning about the respective SDH on bipolar, interval scales (1-to-6) ranging from 'rather important' (1) to 'rather unimportant' (6).

### Knowledge Assessment

The knowledge assessment consisted of an objective assessment and a subjective self-assessment.

#### Objective knowledge assessment

The objective knowledge assessment was performed by means of eight multiple-choice questions related to

##### 1. The Alma-Ata Declaration

To assess whether students know the content of the Alma-Ata Declaration the question 'Which statements are correct?' was followed by four different answer options, of which only one was correctly describing the content of the declaration (Additional File 1: Annex 1). Knowledge of the Alma-Ata Declaration was applied as an indicator to estimate indirectly whether or not students have dealt in-depth with the primary health care concept of the World Health Organisation.

##### 2. Poverty definitions

To assess whether students know common definitions of absolute and relative poverty, the question 'Which definition is correct?' was followed by four answer options, of which only one offered correct definitions of absolute or relative poverty. (Additional File 1: Annex 1)

##### 3. Trends in global fertility and life-expectancy

To assess students' knowledge of trends in global fertility and life-expectancy, we used a 1-to-6 scale (1 = rather correct; 6 = rather incorrect) and captured students' agreement to the following statement: 'Small families and a high life expectancy are characteristic for industrialised countries and large families with a low life expectancy are characteristic for developing countries'. (Additional File 1: Annex 1)

##### 4. Under-five mortality rates of different country-pairs

It is known that under-five mortality rates (U5MR) are associated with the general performance of a health system and with social determinants of health such as women's education, food availability and housing conditions. By assessing students' knowledge of U5MR of different country pairs, we aimed to indirectly assess students' knowledge of the associated determinants in the countries of interest. Students were not asked to provide absolute figures of U5MR, but were rather asked 'Which country among the following country pairs has the higher U5MR?' A set of five country pairs was subsequently provided and students were asked to tick the country which - in their opinion - had a higher U5MR among each pair. Each correct answer was granted with one score, leading to a maximum of five achievable scores in this question. (Additional File 1: Annex 1)

A maximum of eight scores could be achieved by answering all questions in the objective knowledge assessment correctly.

#### Subjective knowledge assessment

Students could also perform a self-assessment of their own knowledge of global health issues. For this purpose we used an operationalised definition of 'global health' to clarify the topics which deem to fall under an according subject heading. We defined 'global health' as an area 'in which students analyse the influence on people's health of factors such as poverty, debt, globalisation, health systems and health financing, human rights, hunger, armed conflicts and migration'. Students were then asked to assess their own knowledge related to these issues on a 1-to-6 scale (1 = rather good; 6 = rather bad). To compare different ratings between subgroups we summarised the answer options 2 to 5 of the 1-to-6 scale to a 'midsection'. This procedure yielded three ordinal variables to express students' subjective knowledge, namely the extrema ('1 = rather important', '6 = rather unimportant') and the summarised midsection ('2-5 = midsection').

### Stratification

To analyse whether the importance placed to the selected SDH as well as students' performance in the knowledge assessment is associated with i) international health electives, ii) education in tropical medicine, or iii) education in global health we used the same stratification criteria regarding students' mobility patterns and their participation in courses of tropical medicine or global health as presented in Table 1 in the first part of this series [4].

### Statistical Analysis

The descriptive analysis of interval variables was performed by calculating means (M), standard deviations (SD), range (min, max), lower- and upper-quartiles (Q_25_, Q_75_) and the median (Mdn).

The distribution of categorical and ordinal data is described with absolute and relative frequency. The differences in distributions of categorical data between independent subgroups were analysed with Fischer's exact test due to the small sample sizes of subgroups. To analyse the distribution of values on interval scales the Kolmogorov-Smirnov test was applied. To compare two independent samples for which a normal distribution could not be assumed, we performed the Mann-Whitney U test. To compare more than two independent samples for which a normal distribution could not be assumed the Kruskall-Wallis-Test was applied.

All tests were performed two-tailed; the level of significance was set at α = 0.05. Analyses were done with SAS version 9.1. and SPSS version 18.0, graphs were additionally created with Microsoft Excel and Adobe Illustrator.

## Results

Students from all 36 medical schools replied [4], resulting in N = 1126 filled-out online-questionnaires. This constitutes an overall response rate of 1.4% from all medical students enrolled during the winter term 2007/2008 in Germany (N = 78.067) [20]. The baseline characteristics of our sample, students' university affiliation, their mobility patterns and their exposure to educational interventions have been described previously [4]. All data and p-values are attached in separate tables in Annexes 2 - 7 as additional file (Additional File 1: Annexes 2-7).

### The importance placed on learning about the social determinants of health

**Figure 1:**
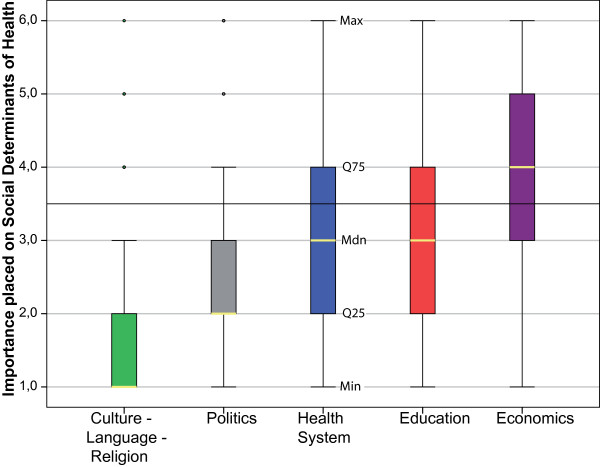
**Importance placed on the social determinants of health by all students (N = 1126)**. 1 = rather important; 6 = rather unimportant. Min = minima; Max = maxima; Q25/Q75 = lower/upper quartile; Mdn = median; o = outliers.

**Figure 2:**
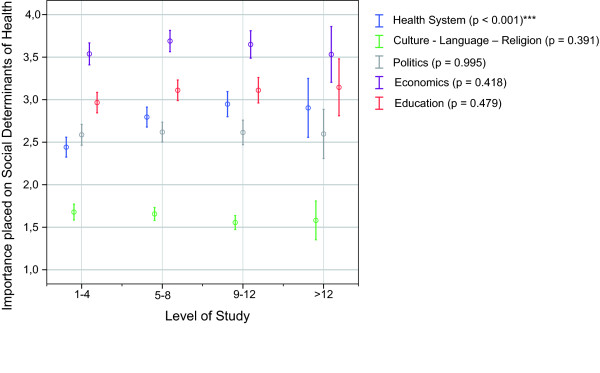
**Importance placed on the social determinants of health by level of study**. 1 = rather important; 6 = rather unimportant; Mean ratings (95.0% CI) p-values of Kruskall-Wallis-Test; ***extremely significant (p ≤ 0.001); 1-4: 1^st ^and 2^nd ^year students; 5-8: 3^rd ^and 4^th ^year students; 9-12: 5^th ^and 6^th ^year students; > 12: above 12 terms of study.

**Figure 3:**
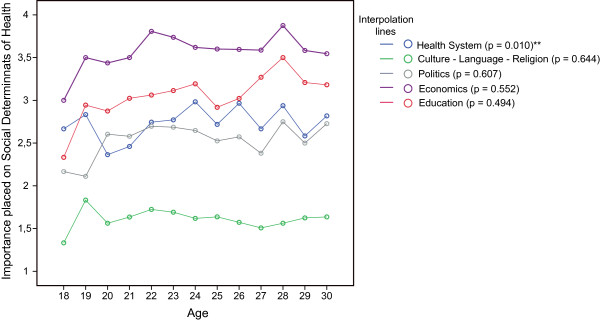
**Importance placed on the social determinants of health by age**. 1 = rather important; 6 = rather unimportant; Mean ratings interpolated p-values of Kruskall-Wallis-Test; **highly significant (p ≤ 0.01).

We did not assume a normal distribution for students' ratings of the SDH (p < 0.001) (Additional File 1: Annex 2). The importance placed on learning about the different SDH varied considerably between the different items. Considering the median, medical students rated learning about the 'economic system' as least important, while 'culture, language and religion' was placed the highest importance (Figure 1). The influence of the level of study evolved to be statistically not significant regarding the importance placed on respective items, except for the item 'health system structure'. First and 2^nd ^year students significantly placed a higher importance (p < 0.001) on learning about the 'health system structure' than students in higher terms (Figure 2). Although younger students appeared to place a higher importance on the SDH in general, the influence of age was statistically not significant with exception for the item 'health system structure'. Students aged 20 and 21 placed a higher importance on this item than both their older and younger colleagues. (Figure 3)

#### i) Social determinants of health by destination countries of international health electives

**Figure 4:**
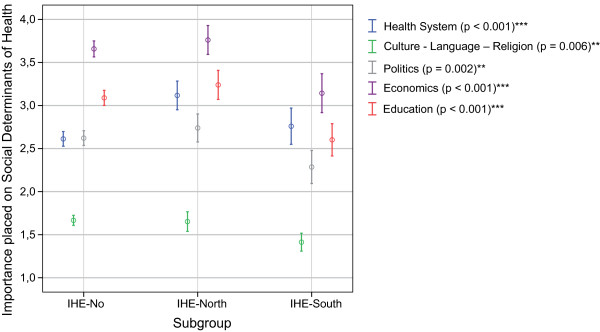
**Importance placed on the social determinants of health by destination of international health electives**. 1 = rather important; 6 = rather unimportant; Mean ratings (95.0% CI) p-values of Kruskall-Wallis-Test; **highly significant (p ≤ 0.01); ***extremely significant (p ≤ 0.001); IHE-South: electives predominantly in developing countries; IHE-North: electives predominantly in industrialised countries; IHE-No: no electives completed abroad.

Learning about all provided social determinants of health was rated significantly higher by students who had previously completed their international health electives (IHE) predominantly in developing countries (IHE-South) compared to students who completed their IHE predominantly in industrialised countries (IHE-North) or had no experiences abroad (IHE-no). In line with the findings above, the only exception was the item 'health system structure', which was rated significantly higher (p < 0.001) by students without experiences abroad (IHE-no) (Figure 4).

#### ii) Social determinants of health by participation in courses of tropical medicine

**Figure 5:**
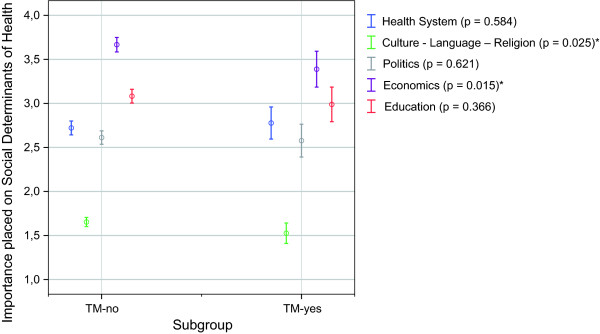
**Importance placed on the social determinants of health by participation in courses of tropical medicine**. 1 = rather important; 6 = rather unimportant; Mean ratings (95.0% CI) p-values of Mann-Whitney-U Test; *significant (p ≤ 0.05); **highly significant (p ≤ 0.01); ***extremely significant (p ≤ 0.001); TM-yes: declared a participation in tropical medicine courses; TM-no: declared no participation in tropical medicine courses.

Respondents who had previously participated in courses of tropical medicine (TM-yes) rated learning about the 'economic system' and about 'culture, language and religion' significantly higher than respondents who had never completed a course in tropical medicine (TM-no) (Figure 5). The importance placed on other items was statistically not significantly different between the two cohorts.

#### iii) Social determinants of health by participation in courses of global health

**Figure 6:**
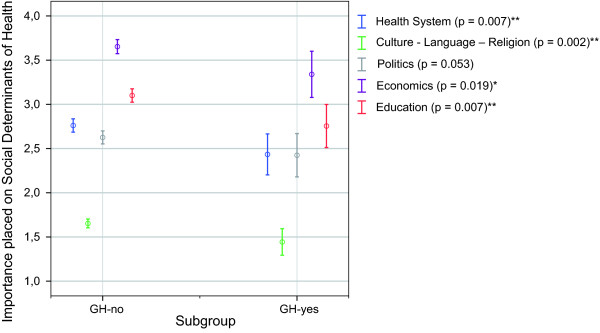
**Importance placed on the social determinants of health by participation in courses of global health**. 1 = rather important; 6 = rather unimportant; Mean ratings (95.0% CI) p-values of Mann-Whitney-U Test; *significant (p ≤ 0.05); **highly significant (p ≤ 0.01); ***extremely significant (p ≤ 0.001); GH-yes: declared a participation in global health courses; GH-no: declared no participation in global health courses.

Similar to the comparison between the cohorts 'TM-yes' and 'TM-no', respondents who had participated in courses of global health (GH-yes) rated learning about the 'economic system' as well as 'culture, language and religion' significantly higher than respondents who had never completed courses in global health (GH-no). The 'GH-yes' cohort additionally placed a significantly higher importance on learning about the 'educational system' and the 'health system structure' than the according comparison group (GH-no) (Figure 6). For the item 'politics', the critical p-value of the Mann-Whitney U Test was marginally exceeded (p = 0.053) so that the differences between the cohorts were statistically not significant.

It is noteworthy that for participants in global health (GH-yes) and tropical medicine (TM-yes), we found a slight overlap between the cohorts. As we have shown previously, 22.9% (40/175) of all students who participated in courses of tropical medicine, also participated in a global health course [4]. Beyond the slight overlap the cohorts were not identical and represented different subgroups.

### Knowledge Assessment

This sections reports descriptively about the results of the knowledge assessment.

#### Objective knowledge assessment

Students achieved an average score of 3.6 (SD 1.5) out of a maximum score of 8.0; 75.0% of our sample achieved a score of 4.0 or less (Q_75 _= 4.0). (Table 1)

**Table 1:** Descriptive results of objective knowledge assessment

Achieved scores in the multiple choice test by all students								
	**Variable**							

**Results of**	**N**	**Mean Score**	**SD**	**Min**	**Q**_**25**_	**Mdn**	**Q**_**75**_	**Max**

All questions	1126	3.6	1.5	0.0	3.0	4.0	4.0	8.0

U5MR questions	1126	1.9	1.2	0.0	1.0	2.0	3.0	5.0

##### 1. The Alma-Ata Declaration

**Figure 7:**
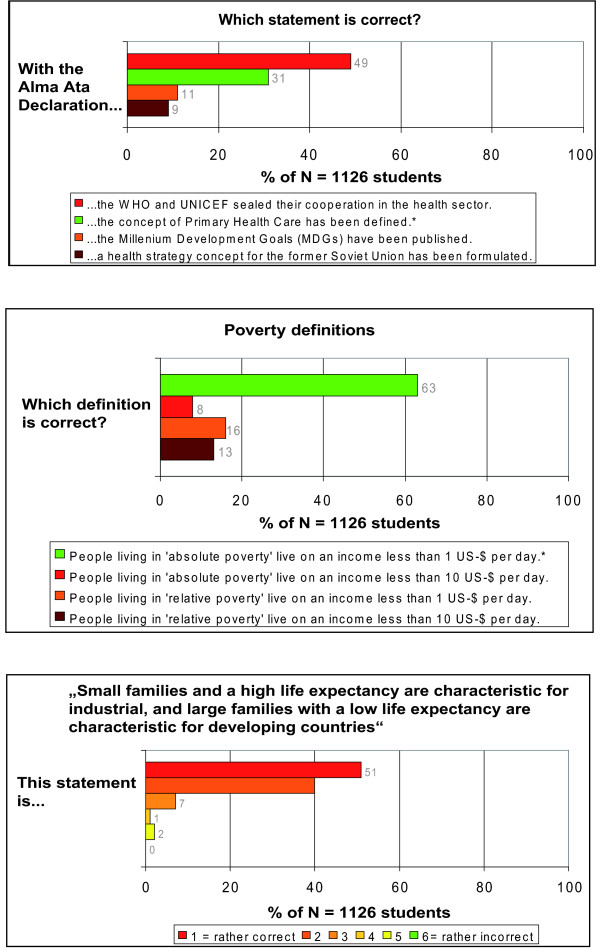
**Distribution of answers in the objective knowledge assessment**. *Answers accepted as correct answers.

69.0% (n = 779) of all respondents did not know the correct answer related to the content of the Alma-Ata Declaration, while only 31.0% (n = 347) stated correctly that the concept of Primary Health Care has been defined within the declaration. (Figure 7)

##### 2. Poverty definitions

The 1 US-Dollar per day definition of absolute poverty according to the income definition of the World Bank was identified correctly by 63.0% (n = 710), while 37.0% (416) chose wrong definitions of absolute and relative poverty. (Figure 7)

##### 3. Trends in global fertility and life-expectancy

98.0% of the respondents chose one of the first three items of the 1-to-6 scale related to the statement of trends in world demographics, showing a high level of agreement to the provided statement. Only four respondents (0.0%) did not agree at all with the statement and declared that the provided statement was 'rather incorrect'. (Figure 7)

##### 4. Under-five mortality rates (U5MR) of five country-pairs

From five achievable scores in this question, students achieved an average score of 1.9 (SD 1.2) (Table 1). This score is below the average score of 2.5 which we could expect if students had randomly chosen five countries.

**Figure 8:**
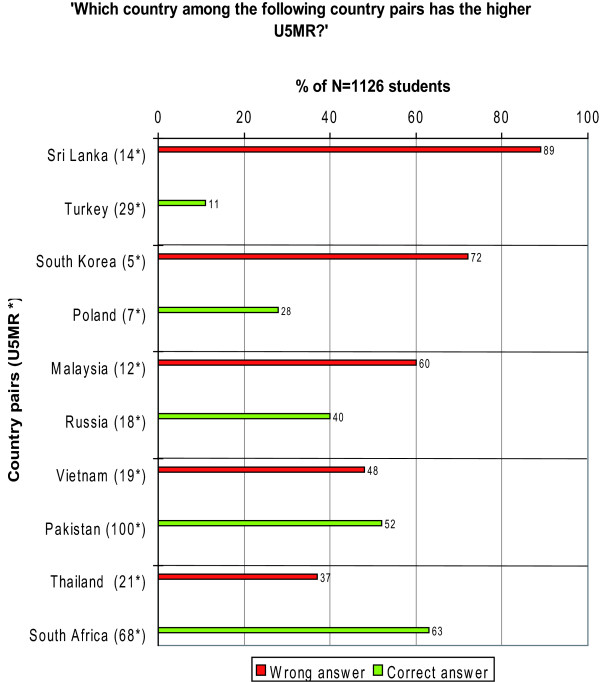
**Distribution of answers related to under-five mortality rates (U5MR) of five country pairs**. * U5MR = Under-five mortality rate; the probability of dying between birth and exactly five years of age expressed per 1,000 live births. Data sources: World Health Statistics 2007.WHO; The State of the World's children 2007. UNICEF, WHO, UN. Population Division and United Nations Statistics Division.

For the country pairs 'Sri Lanka/Turkey', 'South Korea/Poland' and 'Malaysia/Russia' the majority of students falsely declared the first country of each pair as being the country with a higher U5MR, although this was not the case. We received only slight differences in the distribution of responses for 'Vietnam/Pakistan', although these two countries had the highest difference between their U5MR compared to all other country pairs. Only for the country pair 'Thailand/South Africa' the majority correctly chose South Africa as being the country with the higher U5MR. (Figure 8)

#### Subjective knowledge assessment

**Figure 9:**
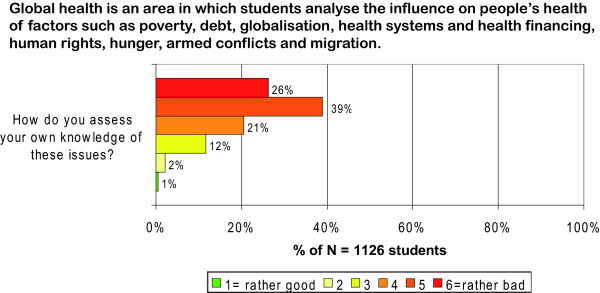
**Distribution of answers in the subjective knowledge assessment**.

26.0% of all students assessed their own knowledge on 'global health' issues explicitly as 'rather bad'; 86.0% of the responses were distributed among the answer options 4, 5 and 6 of the 1-to-6 scale. 15.0% were distributed among the answer options 1, 2 and 3 of the 1-to-6 scale, while only 1.0% of the students explicitly assessed their knowledge on global health issues as 'rather good'. (Figure 9)

### Students' performance in the knowledge assessment by subgroups

This section reports about the association of students' performance in the knowledge assessment with i) students' mobility patterns and participation in courses of ii) tropical medicine and iii) global health.

#### Performance in objective knowledge assessment by subgroups

##### i) Performance by students' mobility patterns

**Figure 10:**
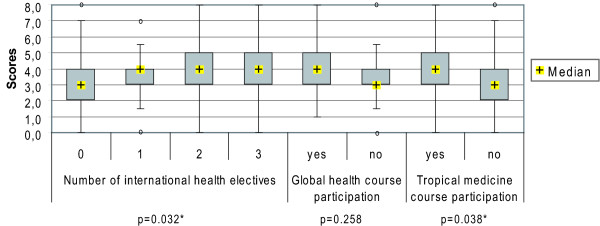
**Students' performance in objective knowledge assessment by subgroups**. p-values of Mann-Whitney U test; *significant (p ≤ 0.05); o = outliers.

In the first part of this series we have reported about the mobility patterns of our sample and the differences in the amount of completed electives, which ranged from 'no electives completed abroad' to three electives completed abroad [4]. Higher scores in the objective knowledge assessment were observed to be significantly (p = 0.032) associated with a higher amount of completed international health electives (IHE). The more often students had been abroad, the higher were the average score, the median and the upper quartile. (Figure 10)

##### ii) Performance by participation in tropical medicine courses

16.0% of our sample had already completed a course in tropical medicine [4]. These students achieved a significantly (p = 0.038) higher score in the knowledge assessment than students who had never participated in a course of tropical medicine, although the assessment contained no questions directly related to tropical medicine and infectious diseases. (Figure 10)

##### iii) Performance by participation in global health courses

9.0% of our sample had already participated at least once in a global health course of undetermined length [4]. Although the descriptive performance of students who had previously been exposed to courses in global health was better than the respective comparison group who had never participated in a global health course, the performance of these students was not significantly (p = 0.258) better than the respective comparison group. The scores of this groups were descriptively similar to that of the cohort of students who had participated in courses of tropical medicine. (Figure 10)

The descriptive data and respective p-values for each subgroup are listed in Annex 7 as additional file (Additional File 1: Annex 7)

#### Subjective knowledge assessment by subgroups

Students who had already completed courses in tropical medicine (p = 0.005) or global health (p = 0.0001) assessed their own knowledge in global health issues significantly higher on the 1-to-6 scale than students who had never participated in these courses. The higher respondents rated their own knowledge, the higher was the participation rate in courses of tropical medicine or global health. (Table 2)

**Table 2:** Subjective assessment of knowledge of global health issues by course participation

Subjective assessment of knowledge of global health issues by participation in courses of global health or tropical medicine						
		**Course participation**				
		
		**yes**		**no**		

**Course**	**Self-assessment of knowledge on 1-to-6 scale**	**Absolute freq.**	%*	**Absolute freq.**	%*	**p-value**^**§**^

Global Health	rather good (1)	2	33.3	4	66.7	0.0001***
		
	midsection (2-5)	92	11.2	733	88.9	
		
	rather bad (6)	12	4.1	283	95.9	


Tropical Medicine	rather good (1)	3	50.0	3	50.0	0.005**
		
	midsection (2-5)	139	16.9	686	83.2	
		
	rather bad (6)	33	11.2	262	88.8	

*Percentages refer to proportions of "Course participation yes/no" among the respective response categories (line total = 100%). ^§^p-value of Fisher's exact test; **highly significant; ***extremely significant

## Discussion

The purpose of this study was to assess the importance medical students place on learning about selected social determinants of health (SDH) and to examine their knowledge of selected global health topics. We further aimed to analyse the interrelation of both of the above factors with i) students' mobility patterns, ii) education in tropical medicine and iii) education in global health.

We have shown that, in relative terms, learning about important social determinants of health (SDH) such as the economic system is given a lower value by students in our sample than learning about determinants related to the cultural context, the health system, educational system or general politics (Figure 1) of a country.

We found a strong relationship between the importance placed on learning about the SDH and factors such as students' mobility and previous exposure to educational interventions. Our results show that students who completed their IHE predominantly in developing countries rate the importance of learning about all examined SDH significantly higher than students who completed their IHE predominantly in industrialised countries or who had not been abroad yet (Figure 4). Notably, the importance placed on learning about the SDH also correlates with a previous exposure to educational interventions, such as education in tropical medicine or global health. This fact is demonstrated in two ways:

1. Determinants which received relatively low credits such as the 'economic system' (Figure 1) were given a significantly higher importance by the exposed cohorts (TM-yes; GH-yes) than by students who were not exposed to the analysed educational interventions (Figure 5 and Figure 6). Both cohorts who received the respective interventions showed also a significantly higher cultural sensitivity than the comparison group without the given intervention (Figure 5 and Figure 6).

2. Participants of global health courses additionally rated learning about the 'educational system' and the 'health system structure' significantly higher than the respective cohort who was not exposed to the analysed courses. (Figure 5 and Figure 6)

A reason for this difference could be, that tropical diseases from a western view are often regarded as poverty-related diseases and that courses of tropical medicine - if any aspects beyond clinical issues are considered - mainly focus on public health problems and poverty related problems in developing countries. In contrast, global health discusses health challenges under a more comprehensive context, which emphasises the importance of various influences on health, not only economic ones. Surprisingly, the only item which was not rated significantly higher by the GH-yes cohort was 'politics'. The smaller sample size of the course participants (GH-yes: n = 106) produced an overall broader confidence interval than for the non-participants (GH-no: n = 1020) and resulted in overlaps if the ratings did not differ by much. For 'politics', this fact produced the marginally significant p-value, which might have been statistically significant with a larger sample size of course participants (GH-yes).

Given the fact that the proportion of 3^rd ^and 4^th ^as well as 5^th ^and 6^th ^years students was considerably higher among the subgroups who gave a higher importance to SDH (i.e. IHE-yes, GH-yes, TM-yes) [4], one could argue that age and level of study could be confounders of the identified differences. However, we have shown that increasing age and level of study as possible confounders did not significantly add to students' interest in learning about SDH (Figure 2 and Figure 3). On the contrary, younger students rather seemed to place a higher importance to SDH than older students. In fact, younger students (aged 20 and 21) and 1^st ^and 2^nd ^year students, who constituted a higher proportion of those without experiences abroad (IHE-no) [4], appeared to be quite 'health system-centred' as far as their interest in SDH was concerned. We believe this might indicate a lower sensitivity for other influences on health.

The phenomenon that medical students' socio-political views are more conservative by the time they finish medical school than upon entrance has been described earlier in the literature [21]. These changes in students' attitudes have been attributed to their personal background characteristics rather than to the characteristics of their professional medical training [21]. We have not captured enough personal characteristics (e.g. gender, cultural background, socio-economic status) to further analyse their influence as confounders on students' attitudes towards SDH in our sample. But we note that the influence of professional medical training on students' attitudes towards learning about SDH is not neglectable. While increasing age and level of study did not correlate with increasing interest in learning about SDH, this correlation worked well especially for international rotations to developing countries, courses of global health and - partially - for courses of tropical medicine. Via these courses and exposures students might have learnt that a society's health depends on more than the 'health system structure', and might thus have appreciated learning about the influences of factors beyond the health care sector more than colleagues without similar exposure.

The results of our study thus lend further credence to Thompson et al., whose paper summarises the benefits of an international health track for US and Canadian students as reported by participants, in terms of 'improved understanding of the importance of public health' and 'increased cultural competency' [22]. In our study, we additionally distinguished between international health tracks in developing and industrialised countries and analysed more detailed the importance placed on *different *factors, which influence public health in terms of the social determinants of health. Our findings further indicate that cultural sensitivity and an understanding of the importance of the SDH is not only associated with IHE, but also with the exposure to courses in tropical medicine or global health.

Whether or not these learning experiences also sensitise students to the influences of SDH within *their own *country and how these experiences might impact on their work as health professionals remains central to future studies on the subject. Experiences from the UK, however, indicate that students benefit from structured courses in this context [23,24]. Students who have undertaken special study modules in global health issues and have learnt about the SDH beyond domestic boundaries have not only found having to think laterally refreshing [23]. They also felt that this kind of learning gave them 'insight into issues that were relevant to practising medicine at home: the health problems faced by asylum seekers and others migrating to the UK; the strength of interest groups such as the pharmaceutical industry; and international trade rules that might affect provision of health care in the UK as well as overseas.' [24]

The results of our knowledge assessment underline the importance of structured teaching in this context. As far as students' knowledge of selected global health issues is concerned, we note that students' performance in objective knowledge was below average (M = 3.6) and in our opinion totally inadequate. 75.0% of our sample achieved a score which was equal or less than 50.0% of the maximum achievable score (Table 1).

The fact that 69.0% of the respondents did not have the vaguest notion about the content of the Alma-Ata Declaration [25] is in our opinion appalling and indicates that students have not dealt in-depth with the primary health care strategy. This strategy embraces promotive, preventive, curative and rehabilitative care [7] and is also relevant for health and health policies in Germany [26]. In the last 30 years, the German health system has experienced an 18.0% decline in the proportion of primary-care doctors (from 65.4% in 1979 to 47.6% in 2009), while the proportion of specialised and superspecialised doctors has increased accordingly (52.4% in 2009) [27]. Thus, the German Medical Association (*Bundesärztekammer*) has recently referred to this relationship as a strongly imbalanced one [27]. In view of our findings, we regard attempts to strengthen primary-care without considering to revitalise the principles and values of primary health care [7] through a corresponding response from *medical education *as highly questionable; a fact which has been noted by the WHO more than 15 years ago [28,29].

Moreover, the results of the questions related to the under-five mortality rates of different country-pairs indicate a biased world-view among our sample towards 'the West and the rest'. As for country pairs, which consisted of both a 'not-European' country and a 'European' country, the vast majority of students declared the 'not-European' country falsely as the one with the higher U5MR indicating preconceived ideas about health in 'not-European' countries. This pattern was coherently found for the country pairs 'Sri Lanka/Turkey', 'South Korea/Poland' and 'Malaysia/Russia' (Figure 8), despite the fact that the 'not-European' counterparts always had the lower U5MR.

If the pair consisted of two 'not-European' countries ('Vietnam/Pakistan') a nearly similar distribution was received as long as no African country was involved. This distribution can be interpreted as a random or indifferent choice made by students between the two 'not-European' countries - despite the high gradient in U5MR between Vietnam and Pakistan (Figure 8). As soon as an African country was involved in the country pairs ('Thailand/South Africa') the choice was clearly (and in this case correctly) in favour of the African country (Figure 8). The overall results of the questions related to U5MR (Table 1) are thus very similar to those produced by a sample of Swedish students, where preconceived ideas were supposed to be the reason for students' response patterns [30].

The assumption prevalent among some experts in international and global health that students perceive global health issues in 'terms of black and white' is also confirmed by the results of the question related to trends in global fertility and life-expectancy (Figure 7). 98.0% of the respondents agreed with the statement, that 'small families and a high life expectancy are characteristic of industrialised countries and large families with a low life expectancy are characteristic of developing countries'. They thus neglect the decline in fertility and the gains in life-expectancy [31] which have been achieved globally in the last decades - also in 'developing countries'; except for Sub-Saharan Africa, Central and Eastern Europe and the Commonwealth of Independent States where life-expectancy is on decline [32]. Furthermore, the high level of agreement to this statement raises questions about students' knowledge of the huge disparities in fertility and life-expectancy that can be found globally along the social gradient within countries - irrespective of the label 'industrialised' or 'developing' country.

The question with the highest proportion of correct answers was the one related to poverty definitions (Figure 7), but still 37.0% of the respondents knew neither the simple income definition of absolute poverty nor the correct definitions of relative poverty. It is not our purpose here to discuss the appropriateness of the 1 US-$ poverty line to define absolute poverty, but we believe it is astonishing, that such a high proportion of medical students has obviously not even cursorily dealt with poverty issues to identify this income definition of absolute poverty among the provided answer options. Given the fact that 15% of children below 15 years in the year 2003 lived at risk of relative poverty in Germany (i.e. lived in families on an income less than 60% of national median equivalised household income) [33], we strongly wonder to what extent the students of our sample are aware of this fact - or of the entailed health inequities that can evidently be found among school children in Germany with low socio-economic status [34].

The subjective knowledge assessment shows that the knowledge gaps among students in our sample are not only assumed by us, but that students themselves perceive knowledge gaps related to the 'influence of factors such as poverty, debts, globalisation, health systems and health financing, human rights, hunger, armed conflicts and migration on people's health' (Figure 9). The subjective assessments are thus consistent with the results of the objective assessment and indicate educational deficits regarding global health issues.

It is of note that students' performance in the knowledge assessment was significantly associated both with students' mobility and with education in tropical medicine (Figure 10). Although the association was not significant for education in global health, in descriptive terms this subgroup had the best performance since it had the highest minimum score accompanied by higher or equal results for the mean and the median compared to the other subgroups (Additional File 1: Annex 7). It seems likely that the lack of statistical significance is due to the smaller sample size of the respective cohort of global health course participants.

The strong association of students' subjective knowledge with courses in tropical medicine and - even more significantly - global health (Table 2) marks these areas out as interventions to respond to the educational deficits.

In this context, one might argue that students who had completed a course in tropical medicine or global health, did not raise their absolute scores in objective knowledge by much despite scoring statistically or descriptively higher. However, it should be considered that with a reference line of a maximum of 8.0 achievable scores, an increase in only one score is already a 12.5% increase in performance.

In addition, due to the lack of a national consensus on learning outcomes in tropical medicine or global health, the authors restrain themselves from concluding whether the courses to which students were exposed achieved their learning objectives or not. We assume that the reason course participants did not raise their absolute scores by much is that the courses did not solely focus on the areas tested in our assessment, but might have aimed to reach other learning outcomes.

It is important to note that both tropical medicine and global health are far from being compulsory courses in contemporary German medical education. Consequently, the disciplines which can be held accountable to impart the tested areas are mainly socio-medical subjects, such as medical sociology in pre-clinical studies as well as social medicine and the cross-sectional subjects, which cover epidemiology, health economics, public health, prevention and health promotion during clinical studies [12]. Issues related to trends in world demographics are explicitly listed in the subject catalogues of socio-medical subjects in both pre-clinicial [35] and clinical terms [36]. While poverty issues are mentioned in syllabi of medical sociology at least in the context of 'infant mortality' and 'living conditions of the elderly' [35], the socio-medical catalogues in clinical terms do not explicitly mention the term 'poverty' [36]. Similarly, neither the Alma-Ata Declaration nor the concept of primary health care as a strategy for health promotion are explicitly listed in pre-clinical [35] or clinical [36] subject catalogues. The poor results of the knowledge assessment indicate that for the tested areas, the socio-medical courses either did not work or did not intend to work - as indicated by the absence of the tested topics in respective syllabi.

Further research and in-depth knowledge assessments are certainly necessary to draw more valid conclusions on students' understanding of public health issues on a national or global dimension.

## Limitations and strengths

The study design applied to answer the research questions bears several weaknesses. Recruiting participants by using electronic mailing-list of students' unions bears the possibility that individuals responded more than once to the survey, e.g. if the student was on multiple lists. The survey software we used had no option to avoid this without conflicting with the anonymity of the survey.

Furthermore, the recruitment methodology attempted to reach as many students as possible. Therefore we received an opportunistic sample and can determine response rates (1.4%) only related to the whole population of medical students in Germany (78.067). Consequently, the findings presented in this paper cannot be generalised. Additionally, we have only received substantial student responses (above 50) from a few medical schools [4]. Therefore, any conclusions refer strictly to the described sample. Our findings do not allow conclusions about individual schools, but provide some indication of trends which should be analysed further on local levels.

Recruiting participants via mailing-list of students' unions may imply selection bias towards 'especially motivated' students. In this case however, the importance 'unmotivated' students place to learning about the SDH would most probably be even more alarming, as already in our sample important determinants such as the 'economic system' received relatively low ratings only; not to mention the potential performance of 'unmotivated' students in the knowledge assessment.

We also note that the co-incidences found between the SDH and IHE or educational interventions, such as education in global health or tropical medicine, do not allow us to make any statements about *cause and effect*. Thus we cannot determine whether the higher importance placed on learning about the SDH is a cause or effect of completing an IHE in developing countries or of participating in a course of tropical medicine or global health. However, beyond debates on cause and effect we believe that even if schemes and interventions simply serve to reinforce previous motivation (e.g. of younger students), that outcome should not be dismissed [37]. Rather, it should be used to enhance the full potential of synergy effects.

Furthermore, the question which captured the importance placed on learning about the SDH explicitly referred to learning about SDH in countries other than Germany. Therefore the findings can not be easily transferred to learning about SDH in general or in a national context without further research applying a more general approach. However, we believe it is plausible that factors which reinforce an understanding of the importance of SDH do not distinguish a domestic social space from an international or global one, but rather reinforce a general - or universal - understanding and awareness of the importance of the SDH. To be able to draw better conclusions on students' interest in learning about SDH or the factors which reinforce such an interest, a more qualitative approach could reveal better insights than our quantitative approach. Especially against the background that, despite statistical significances, the differences in rating the importance of SDH between examined subgroups did not always vary by much regarding the mean (95. 0% CI).

Another weakness is reflected in the fact, that the multiple-choice questions we used for the knowledge assessment were not validated or pre-tested in advance, except for the questions related to U5MR, which have been previously performed on Swedish students [30]. The amount of questions in the knowledge assessment may have been too few to fully or more comprehensively assess knowledge gaps among the sample. However, making the test much longer may have significantly reduced the number of respondents.

Finally, the stratification criteria applied to group medical students according to their participation in courses of global health or tropical medicine, could have been better specified in terms of the timeframe, the providers and especially the content of the respective courses.

Despite the low response rate (1.4%), we received a sufficiently large number of responses (N = 1126) to draw conclusions among our sample about knowledge gaps and the correlation of the examined factors on the importance placed to the SDH. We further believe the conclusions from this sample might be more comprehensive than from one with higher relative size, but considerably smaller absolute size. Previous studies which analysed various outcomes of IHE included only samples with relatively small absolute size (12 to maximum 192 students) and mostly used non-validated self-reported questionnaires [22]. There are no studies known to us dealing with similar questions or with students' knowledge of international or global health issues with regard to Germany *(Bozorgmehr K, Tinnemann P: The State of Global Health in German Medical Education: a systematic review, unpublished)*. Thus, with this first analysis of German medical students' attitude towards learning about SDH and with the assessment of their knowledge of health issues beyond national boundaries and beyond bio-medical topics, we have filled some evidence in existing research gaps *(ibid., unpublished)*. Despite the limitations of our approach, we hope that this work provides a basis and motivation for future research on outcomes, deficits and potential benefits related to global health education.

## Implications and Recommendations

In a narrow sense, the low participation rate in courses of tropical medicine among our sample [4] calls for an up-scaling of international health in German medical education. In one sense, tropical diseases are no different than other infectious diseases: they spread. Learning about tropical medicine should be compulsory for all medical students, but teaching tropical medicine must also consider the broader social context of the diseases. We believe that it is very important for all students to know about tropical diseases not only to provide better care for immigrants and travellers as usually stated from a "western" view. We regard knowledge about tropical diseases *and *their social aetiology as a pre-requisite to act on and react to internationally and globally interacting links between diseases and their social determinants. In particular, we believe that research and resources for neglected diseases, access to medicines, patents and intellectual property rights as well as health workforce migration and 'brain gain' by industrialised countries are just a few examples of issues on which medical students, health professionals and universities can take local action with an international impact, making it necessary to include these issues in the education of health professionals.

In a broader sense, huge health inequalities and disparities exist between and within countries [6] and dismantle any clear-cut distinction between "developing" and "developed" world. The need to learn about the social determinants of health (SDH) is thus not only important for students who take part in international health electives to developing countries, but also for the majority of students in our sample who crossed borders within Europe and North America [4].

But how can the recommendation of the Commission on the Social Determinants of Health - to make SDH a 'standard and compulsory part of training of medical and health professionals' - be realised in a manner in which the complexity and 'social science' character of these issues elicits the interest of medical students? Many arguments can be found in the literature against a 'wide' orientation of medical education with a strong emphasis on topics beyond the biomedical field. The following statement by the famous Viennese surgeon Theodor Billroth in 1876 illustrates the fact that discussions about the scope of medical education are not new:

"This discipline [..]", Billroth stated, meaning the "[..] whole social medicine [..]", "[..] will never be of high interest to students, who have got their hands full to cope with the diseases of individuals and have just as little appreciation for the wellbeing of the community as for politics and diplomacy in practice." [38]

On the basis of our findings, we contest this and other generalising statements about students' disinterest in socio-medical fields [14,15], pointing out that many of these statements are based upon aggregated data only.

The sample of students examined in this series expressed a high demand for global health learning opportunities and showed an overall high mobility correlating with dissatisfaction with the existing supply of global health courses [4]. Additionally, we found co-incidences between higher ratings of the importance of learning about the SDH and completion of global health courses and IHE, especially in developing countries.

We believe that all of these factors constitute a window for educational interventions aimed at enforcing an appreciation of learning about the social determinants of health beyond the diseases of individuals. To achieve this objective and to make use of the full potential of IHE, we make the following recommendations to medical schools and public health educators.

### Recommendation 1

To define clear learning objectives for IHE *beyond *curative aspects and provide formal and structured pre-, and post-, elective preparation and trainings at medical schools.

Preparation opportunities should not only include travel health risks nor strictly focus on tropical medicine. We have shown that students are predominantly involved in curative work abroad [4]. A stronger education in tropical medicine, aimed at increasing the appallingly low exposure of students to this subject [4], can certainly prepare for the clinical reality abroad especially in developing countries.

But we feel that this approach alone is insufficient to impart an understanding of the broader determinants of health, especially if the majority of movements take place within Europe, as was the case in our sample [4]. The inclusion of 'global' topics such as the principles of comprehensive primary health care, health system comparisons, root causes of inequities in health as well as international interdependencies of health and health policy issues must be considered in pre-elective preparation courses.

Further attention must be paid to the creation of transnational university partnerships based *on an equal footing *in order to avoid a unidirectional flow of knowledge and benefits (e.g. from developing to industrialised countries). The creation of 'real' partnerships becomes highly important in light of critical voices warning us of old and new forms of exploitation in the name of 'global health' [39,40].

### Recommendation 2

To enable debriefings after IHE and to provide in-depth opportunities as part of medical studies to learn and reflect about the social, economic, political and cultural forces that shape health across the world. These debriefings are important to channel experiences gained abroad - either in developing or equally in industrialised countries. This could be achieved in the form of structured and comprehensive education in global health and may - as we have shown in this paper - cultivate an appreciation for the SDH among medical students. Global health education might cater to the pre-existing interest in political issues found among our sample (Figure 1) and identified among medical students in Germany by others [41]. Further, it might cater to this interest more effectively than conventional approaches to teaching socio-medical issues.

Recommendations with regard to how global health might be embedded in existing curricula in Germany have already been developed by the German Medical Students' Association [42].

### Recommendation 3

To facilitate the transformation of students' experiences and knowledge of SDH into action in order to meet real world requirements and to influence the SDH towards better health for all by formally including action-oriented outcomes in curricula. The evident challenge is to clarify health professionals' role in addressing the SDH - irrespective of their participation in international rotations. Meeting this challenge should gain more attention in research, education and practice.

## Conclusion

We have shown that the importance medical students in our sample place on learning about social determinants of health (SDH) significantly interacts with factors such as mobility and educational interventions. Firstly, we found a positive association between international health electives (IHE) in developing countries and a higher importance placed on learning about the SDH in other countries. Secondly, participation in courses of tropical medicine and global health correlate significantly with a higher interest in learning about the influences of 'culture, language and religion' and of the 'economic system' on health. Global health education correlates additionally with significantly higher importance placed on the 'educational system' and the 'health system structure', while learning about 'politics' in the context of a foreign country was marginally significant. These interrelations should be used as educational windows to cultivate an appreciation for the social determinants of health.

We have provided evidence for knowledge gaps among our sample related to the content of the Alma-Ata Declaration, to global trends in fertility and life expectancy as well as to poverty definitions. Our findings indicate preconceived ideas among medical students in our sample related to the health status of 'not-European' countries. Higher scores in objective knowledge correlated significantly with a higher frequency of IHE and participation in courses of tropical medicine and descriptively with participation in global health courses. The subjective knowledge assessment confirmed the outcomes of the objective knowledge assessment, marking the associated areas out as intervention areas to foster, catalyse and consolidate positive educational outcomes.

We suggest that an adequate inclusion of global health in German medical curricula would not only meet needs and demands among medical students as expressed by our sample [4], but also constitute a highly necessary step to fill existing knowledge gaps, counter simple world-views anchored in past and intellectually comfortable models that are increasingly obsolete, and increase the social relevance of medical education.

Beyond the context of student mobility and pre-elective training, medical schools should regard courses in tropical medicine as a must and consider education in global health as a means to impart an understanding of the social dimension of health. Abroad or at home, the need for health professionals to act on this dimension of health is universal.

## Competing interests

### Financial competing interests

KB, KS and JMS are members of the Globalisation and Health Initiative (GandHI) of the German Medical Students' Association (Bundesvertretung der Medizinstudierenden e.V.). GandHI receives fundings by Capacity Building International (InWEnt)/Federal Ministry for Economic Cooperation and Development (BMZ). This study was financially supported by the German Medical Students' Association. The article-processing charge was covered by the Institute for Social Medicine, Epidemiology and Health Economics, Charité - University Medical Centre Berlin. The authors declare that they have no financial competing interests.

### Non-financial competing interests

This study has been conducted as part of the research thesis of KB to gain an academic degree *(Dr. med) *at the Institute for Social Medicine, Epidemiology and Health Economics, Charité - University Medical Center Berlin, Germany. Although grateful for the financial support by the German Medical Students' Association, the authors attest that the conclusions in this study are theirs alone and not necessarily endorsed by the the Globalisation and Health Initiative (GandHI) or the German Medical Students' Association (Bundesvertretung der Medizinstudierenden e.V.).

## Authors' contributions

All authors have made substantial contributions to the study conception and design. KS and JMS contributed significantly to designing the questionnaire. KB developed the hypotheses, performed the statistical analyses, evaluated and interpreted the findings, produced graphs and tables and drafted and revised the manuscript. PT, KS and JMS reviewed and commented the draft as well as revisions of the same. PT was involved in all steps of the study and provided substantial critical advice during the conduct and evaluation of this study. All authors have read and approved the final manuscript.

## Pre-publication history

The pre-publication history for this paper can be accessed here:

http://www.biomedcentral.com/1472-6920/10/67/prepub

## Supplementary Material

Additional file 1 **Annexes 1-7**. Additional File 1 contains seven tables (Annexes 1-7) related to the questionnaire design in objective knowledge assessment as well as data and p-values related to tests of significance. Click here for file
