# Supplementary material for: Global Health Education: a cross-sectional study among German medical students to identify needs, deficits and potential benefits (Part 2 of 2: Knowledge gaps and potential benefits)
Source: BMC Med Educ. 2010 Oct 8;10:67. doi: 10.1186/1472-6920-10-67 (PMC2958968; doi:10.1186/1472-6920-10-67)
Supplement: Additional file 1 — Annexes 1-7. Additional File 1 contains seven tables (Annexes 1-7) related to the questionnaire design in objective knowledge assessment as well as data and p-values related to tests of significance. [file 1472-6920-10-67-S1.PDF]

ANNEX 1: QUESTIONS AND RESPONSE OPTIONS IN OBJECTIVE KNOWLEDGE ASSESSMENT 2

ANNEX 2: TESTS OF NORMALITY 3

ANNEX 3: IMPORTANCE PLACED ON SOCIAL DETERMINANTS OF HEALTH BY LEVEL OF STUDY – DATA AND P-VALUES 3

ANNEX 4: IMPORTANCE PLACED ON SOCIAL DETERMINANTS OF HEALTH BY DESTINATION OF IHE – DATA AND P-VALUES 4

ANNEX 5: IMPORTANCE PLACED ON SOCIAL DETERMINANTS OF HEALTH BY SUBGROUPS TM-YES/ TM-NO – DATA AND P-VALUES 5

ANNEX 6: IMPORTANCE PLACED ON SOCIAL DETERMINANTS OF HEALTH BY SUBGROUPS GH-YES/ GH-NO – DATA AND P-VALUES 5

ANNEX 7: STUDENTS' PERFORMANCE IN OBJECTIVE KNOWLEDGE ASSESSMENT BY SUBGROUPS – DATA AND P-VALUES 6

Annex 1: Questions and response options in objective knowledge assessment

	**Objective Knowledge Assessment ***
	Which statement is correct? (single choice)
	With the Alma-Ata Declaration…
□	1. a health strategy concept for the former Soviet Union has been formulated.
□	2. the Millenium Development Goals (MDGs) have been published.
□	3. the concept of Primary Health Care has been defined.
□	4. the WHO and UNICEF sealed their cooperation in the health sector.
	Which definition is correct? (single choice)
□	1. People living in 'absolute poverty' live on an income less than 1 US-$ daily.
□	2. People living in 'absolute poverty' live on an income less than 10 US-$ daily.
□	3. People living in 'relative poverty' live on an income less than 1 US-$ daily.
□	4. People living in 'relative poverty' live on an income less than 10 US-$ daily.
	"Small families and low mortality rates are characteristic for industrial, and large families with high mortality rates are characteristic for developing countries"
	This statement is…(single choice)
□	1 = rather correct
□	2
□	3
□	4
□	5
□	6= rather incorrect
	'Which country among the following country pairs has the higher U5MR?' (single choice for each pair)
□	Sri Lanka
	or
□	Turkey
□	South Korea
	or
□	Poland
□	Malaysia
	or
□	Russia
□	Pakistan
	or
□	Vietnam
□	Thailand
	or
□	South Africa

* Questions and answer options of the questionnaire were originally formulated in German language. This table is a translation of the original questions and answer options.

Annex 2: Tests of Normality

Tests of Normality						
	Kolmogorov-Smirnov[a]			Shapiro-Wilk		
	Statistic	df	Sig.	Statistic	df	Sig.
Health System	.196	1126	.000	.914	1126	.000
Culture - Language - Religion	.314	1126	.000	.739	1126	.000
Politics	.226	1126	.000	.898	1126	.000
Economics	.180	1126	.000	.934	1126	.000
Education	.194	1126	.000	.922	1126	.000

a. Lilliefors Significance Correction

Annex 3: Importance placed on social determinants of health by level of study – data and p-values

		Importance placed on Social Determinants of Health							
**Social Determinant of Health**	**Terms**	1 = rather important	2	3	4	5	6 = rather unimportant	**Total**	**p-value[§]**
		Frequency							
Economics	1-4	12	57	118	79	64	21	351	0.418
	5-8	17	61	124	102	77	44	425	
	9-12	13	55	69	69	47	35	288	
	>12	3	10	20	13	12	4	62	
Education	1-4	25	112	104	78	24	8	351	0.479
	5-8	37	106	142	69	55	16	425	
	9-12	22	83	83	57	27	16	288	
	>12	5	15	23	8	7	4	62	
Politics	1-4	56	135	90	42	24	4	351	0.995
	5-8	74	150	110	55	27	9	425	
	9-12	55	96	75	33	25	4	288	
	>12	9	25	14	11	2	1	62	
Health System	1-4	76	121	96	40	17	1	351	<0.001***
	5-8	62	131	113	78	33	8	425	
	9-12	39	75	79	59	29	7	288	
	>12	9	19	16	6	11	1	62	
Culture - Language - Religion	1-4	185	116	32	14	4	0	351	0.391
	5-8	217	152	44	9	3	0	425	
	9-12	161	97	27	3	0	0	288	
	>12	36	20	4	1	0	1	62	

[§]p-value of Kruskall-Wallis-Test, ***extremely significant

Annex 4: Importance placed on social determinants of health by destination of IHE – data and p-values

Social Determinant of Health	International health elective	Importance placed on Social Determinants of Health: 1 = rather important	2	3	4	5	6 = rather unimportant	Total	p-value§
		Frequency							
Economics	IHE-No	28	114	225	184	132	73	756	< 0.001***
	IHE-North	8	31	62	57	51	21	230	
	IHE-South	9	38	43	20	14	9	133	
Education	IHE-No	52	217	231	152	76	28	756	< 0.001***
	IHE-North	18	50	76	44	29	13	230	
	IHE-South	19	48	43	14	8	1	133	
Politics	IHE-No	118	285	198	87	56	12	756	0.002**
	IHE-North	36	75	60	36	18	5	230	
	IHE-South	38	44	31	16	3	1	133	
Health System	IHE-No	140	248	195	119	46	8	756	< 0.001***
	IHE-North	20	62	66	42	33	7	230	
	IHE-South	23	34	42	22	10	2	133	
Culture - Language - Religion	IHE-No	388	262	80	22	4	0	756	0.006**
	IHE-North	122	80	19	5	3	1	230	
	IHE-South	86	39	8	0	0	0	133	

§p-value of Kruskall-Wallis-Test, **highly significant, ***extremely significant

Annex 5: Importance placed on social determinants of health by subgroups TM-yes/ TM-no – data and p-values

Social Determinant of Health	Tropical medicine course	1 = rather important	2	3	4	5	6 = rather unimportant	Total	p-value§
		Importance placed on Social Determinants of Health							
Economics	yes	14	34	50	36	29	12	175	0.015*
	no	31	149	281	227	171	92	951	
Education	yes	24	38	63	24	18	8	175	0.366
	no	65	278	289	188	95	36	951	
Politics	yes	36	57	47	19	12	4	175	0.621
	no	158	349	242	122	66	14	951	
Health System	yes	25	54	52	27	13	4	175	0.584
	no	161	292	252	156	77	13	951	
Culture - Language - Religion	yes	108	47	16	3	1	0	175	0.025*
	no	491	338	91	24	6	1	951	

§p-value of Mann-Whitney U Test, *significant

Annex 6: Importance placed on social determinants of health by subgroups GH-yes/ GH-no – data and p-values

Social Determinant of Health	Global health course	1 = rather important	2	3	4	5	6 = rather unimportant	Total	p-value§
		Importance placed on Social Determinants of Health							
Economics	yes	8	22	34	17	18	7	106	0.019*
	no	37	161	297	246	182	97	1020	
Education	yes	18	29	35	11	11	2	106	0.007**
	no	71	287	317	201	102	42	1020	
Politics	yes	28	35	24	11	5	3	106	0.053
	no	166	371	265	130	73	15	1020	
Health System	yes	26	36	24	13	6	1	106	0.007**
	no	160	310	280	170	84	16	1020	
Culture - Language - Religion	yes	73	23	7	2	1	0	106	0.002**
	no	526	362	100	25	6	1	1020	

§p-value of Mann-Whitney U Test; *significant; **highly significant

Annex 7: Students' performance in objective knowledge assessment by subgroups – data and p-values

Achieved scores in the MC-test depending on participation in courses of GH or tropical medicine										
Course	Participation	N	Mean	SD	Min	Q_{25}	Mdn	Q_{75}	Max	p-value[§]
Global Health	yes	106	3.8	1.5	1.0	3.0	4.0	5.0	8.0	0.258
	no	1020	3.5	1.5	0.0	3.0	3.0	4.0	8.0	
Tropical Medicine	yes	175	3.8	1.6	0.0	3.0	4.0	5.0	8.0	0.038*
	no	951	3.5	1.4	0.0	2.0	3.0	4.0	8.0	

Achieved scores in the MC-test depending on the frequency of international health electives									
Frequency	N	Mean	SD	Min	Q_{25}	Mdn	Q_{75}	Max	p-value[§]
0	756	3.5	1.4	0.0	2.0	3.0	4.0	8.0	
1	221	3.6	1.4	0.0	3.0	4.0	4.0	7.0	
2	92	3.7	1.7	0.0	3.0	4.0	5.0	8.0	
3	57	4.1	1.8	0.0	3.0	4.0	5.0	8.0	
0	756	3.5	1.4	0.0	2.0	3.0	4.0	8.0	0.032*
≥ 1	370	3.7	1.5	0.0	3.0	4.0	5.0	8.0	

[§]p-value of the Mann-Whitney U test; *significant
